# Low Doses of Glyphosate/Roundup Alter Blood–Testis Barrier Integrity in Juvenile Rats

**DOI:** 10.3389/fendo.2021.615678

**Published:** 2021-03-11

**Authors:** Agostina Gorga, Gustavo Marcelo Rindone, Cecilia Lucía Centola, Cristian M. Sobarzo, Eliana Herminia Pellizzari, María del Carmen Camberos, Clara Isabel Marín-Briggiler, Debora J. Cohen, Maria Fernanda Riera, Maria Noel Galardo, Silvina Beatriz Meroni

**Affiliations:** ^1^ Centro de Investigaciones Endocrinológicas “Dr. César Bergadá” (CEDIE) – Consejo Nacional de Investigaciones Científicas y Técnicas (CONICET) – Fundación Endocrinológica Infantil (FEI) – División de Endocrinología, Hospital de Niños Ricardo Gutiérrez, Buenos Aires, Argentina; ^2^ Instituto de Investigaciones Biomédicas (INBIOMED), Consejo Nacional de Investigaciones Científicas y Técnicas (CONICET), Facultad de Medicina, Universidad de Buenos Aires (UBA), Buenos Aires, Argentina; ^3^ Instituto de Biología y Medicina Experimental (IBYME), Consejo Nacional de Investigaciones Científicas y Técnicas (CONICET), Buenos Aires, Argentina

**Keywords:** glyphosate, testis, blood–testis barrier, roundup, male reproduction

## Abstract

It has been postulated that glyphosate (G) or its commercial formulation Roundup (R) might lead to male fertility impairment. In this study, we investigated the possible effects of G or R treatment of juvenile male rats on blood-testis barrier function and on adult male sperm production. Pups were randomly assigned to the following groups: control group (C), receiving water; G2 and G50 groups, receiving 2 and 50 mg/kg/day G respectively; and R2 and R50 groups receiving 2 and 50 mg/kg/day R respectively. Treatments were performed orally from postnatal day (PND) 14 to 30, period of life that is essential to complete a functional blood-testis barrier. Evaluation was done on PND 31. No differences in body and testis weight were observed between groups. Testis histological analysis showed disorganized seminiferous epithelium, with apparent low cellular adhesion in treated animals. Blood-testis barrier permeability to a biotin tracer was examined. A significant increase in permeable tubules was observed in treated groups. To evaluate possible mechanisms that could explain the effects on blood-testis barrier permeability, intratesticular testosterone levels, androgen receptor expression, thiobarbituric acid reactive substances (TBARS) and the expression of intercellular junction proteins (claudin11, occludin, ZO-1, connexin43, 46, and 50 which are components of the blood-testis barrier) were examined. No modifications in the above-mentioned parameters were detected. To evaluate whether juvenile exposure to G and R could have consequences during adulthood, a set of animals of the R50 group was allowed to grow up until PND 90. Histological analysis showed that control and R50 groups had normal cellular associations and complete spermatogenesis. Also, blood-testis barrier function was recovered and testicular weight, daily sperm production, and epididymal sperm motility and morphology did not seem to be modified by juvenile treatment. In conclusion, the results presented herein show that continuous exposure to low doses of G or R alters blood-testis barrier permeability in juvenile rats. However, considering that adult animals treated during the juvenile stage showed no differences in daily sperm production compared with control animals, it is feasible to think that blood-testis barrier impairment is a reversible phenomenon. More studies are needed to determine possible damage in the reproductive function of human juvenile populations exposed to low doses of G or R.

## Introduction

Glyphosate (G)-based herbicides are important tools used worldwide in agriculture, forestry, and weed control (1). In addition, their use have spread because of the development of transgenic plants that tolerate high concentrations of these compounds (2, 3). Roundup (R) is the most widely used formulation. It comprises mixtures of G and adjuvants, such as polyoxyethylene tallowamine (POEA), to enhance the uptake and translocation of the active ingredient into plant cells (4). G prevents plant development by inhibiting the enzyme enolpyruvylshikimate phosphate synthase (EPSPS) and interfering with the synthesis of essential aromatic amino acids (5). As this enzyme is not expressed by any member of the animal kingdom, the actions of G were supposed to be present exclusively in plants (6, 7). However, unexpected effects in the animal kingdom have been observed. Particularly, G might act as an endocrine disruptor which could lead to male fertility impairment. Compelling evidence provide the existence of adverse effects of treatment with G or R on male reproduction (8–12). However, most of them were performed using doses far above the maximum dietary and environmental exposure levels reported in humans. For this reason, whether G is harmful to male reproductive health when exposure occurs at low doses and at early life stage is still under debate.

Sertoli cells provide structural and nutritional support to germ cells. An important and unique physiological function of Sertoli cells is to contribute to the maintenance of a microenvironment suitable for the development of spermatogenesis through the establishment of the blood-testis barrier (BTB) (13, 14). In the rat, BTB begins to be assembled around 15 to 20 days of age when Sertoli cell proliferation ceases. However, a fully functioning BTB is not established until post-natal day (PND) 25 to 30 (15, 16). This period is characterized by the first wave of spermatogenesis up to round spermatids, the presence of numerous large pachytene spermatocytes and the formation of secondary spermatocytes. It is worth mentioning that the first tubules with step 19 spermatids appears on PND 45 and the first spermatozoa in the epididymis appears on PND 52 (17). The presence of an appropriately assembled BTB is essential to maintain spermatogenesis, a process that in the rat takes place during four complete cycles of the seminiferous epithelium and lasts from 49 to 52 days (18). Remarkably, dysfunction of the BTB has been considered an important mechanism involved in xenobiotic-induced reproductive toxicity (19). Recently, in studies using 20-day old rat Sertoli cell cultures, we have shown that G and R treatments alter the Sertoli cell junction barrier and postulated that BTB integrity is a sensitive target for the adverse effects of G or R on male reproductive function (20). Nevertheless, no studies have addressed a possible disruption of BTB by G and/or R treatments *in vivo* and its possible role in reproductive toxicity. In this study we investigated the possible effects of low doses of G and/or R treatment of juvenile rats on BTB function and on adult male sperm production.

## Materials and Methods

### Animals

All the procedures used in this study were approved by the Comité Institucional de Cuidado y Uso de Animales de Laboratorio (CICUAL) from the Hospital de Niños Ricardo Gutiérrez (Res #2018-002) and performed in accordance with the principles and procedures outlined in the Guide for the Care and Use of Laboratory Animals issued by the National Institute of Health of USA. 3-month-old pregnant Sprague Dawley rats (250–300 g) were purchased from the bioterium of the Facultad de Ciencias Veterinarias, Universidad de Buenos Aires (FCV-UBA). In that facility, nulliparous female rats were mated with a stud male. The animals were separated after confirmed copulation either by detection of vaginal plug or spermatozoa in vaginal smear samples. Five days prior to pup delivery, rats were transported to our bioterium in transportation crates and were housed singly in free exchange cages (30 cm x 40 cm x 23 cm) with stainless steel tray containing pine wood shavings as bedding. Animals were maintained under controlled conditions of temperature (20 ± 2°C), relative humidity and lighting (12 h light–12 h dark cycle) with free access to water and pellet laboratory chow (Rat-Mouse Diet, Asociación de Cooperativas Argentinas, Buenos Aires, Argentina).

### Experimental Design

**Figure 1:**
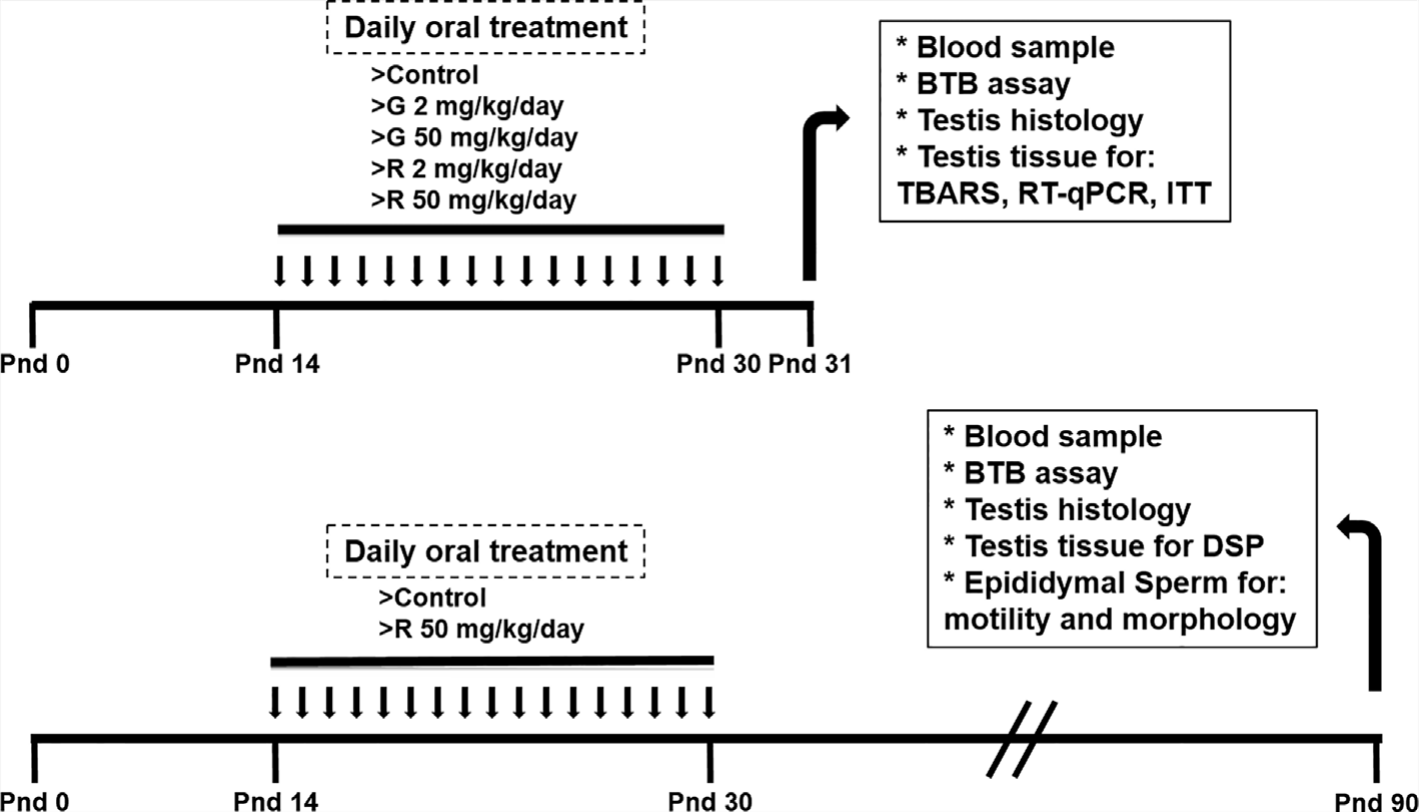
Experimental protocol used to evaluate the effect of different doses of G and R treatment on rats at PND 31 and PND 90.

At delivery, pups were sexed according to the anogenital distance. Litters were adjusted to 8 pups, prioritizing a maximum of 8 male pups per litter when possible. Male pups were randomly assigned to one of the following treatment groups: control group (C), receiving water; G2 and G50 groups, receiving 2 and 50 mg/kg/day G, respectively; and R2 and R50 groups receiving 2 and 50 mg/kg/day R, respectively. G was provided by Sigma-Aldrich (St Louis, USA). R formulation was a liquid water-soluble formulation containing 66.2% of G potassium salt, as its active ingredient. Treatments were given orally from PND 14 to 30. Pups were weaned on PND 21 and euthanized on PND 31. A set of animals were treated from PND 14 to 30 with water or 50 mg/kg/day R and kept without further treatment until PND 90. Tissues and blood were collected from euthanized animals in PND 31 or PND 90 (**Figure 1**).

Rats were treated from PND 14 to 30 to comprise the full period of maturation of the BTB. This exposure period corresponds to Sertoli cell maturation associated with BTB formation, germ cell meiosis, and the appearance of the first spermatids. The dose of 2 mg/kg/day was selected because it is in order of magnitude of the reference dose (RfD) of 1 mg/kg/day recently reassigned for glyphosate by the US EPA (2017). It is worth mentioning that this dose is used in several reports to analyze reproductive toxicity of the herbicide (21–23). The dose of 50 mg/kg/day was selected based on the no observed adverse effect level (NOAEL) for G (24) and on a previous report from Romano et al (10) who proposed this dose of R as appropriate for future toxicological analyses. No alterations in maternal care and nursing among the experimental groups were detected. Treatments caused no overt signs of toxicity.

### Collection of Blood and Tissues

Blood was obtained by intracardiac puncture. The samples were allowed to clot at room temperature for 15 min, and then they were centrifuged at 950*g* for 5 min in order to obtain the serum. Supernatants were immediately frozen at −20°C for subsequent analysis. Biochemical parameters were determined with an automated Cobas c 501 analyzer (Roche Diagnostics, Mannheim, Germany).

At PND 31, animals were euthanized by CO_2_ asphyxiation, and testes were dissected, weighed, and used for histological analysis and BTB assay. Also, testes were dissected and snap frozen for reverse transcription quantitative real-time polymerase chain reaction (RT-qPCR) and Thiobarbituric Acid Reactive Substances (TBARS) determination. Tissue samples (liver, kidney, stomach, and intestine) were also collected at that time.

At PND 90, animals were anesthetized with a mixture of ketamine-xylazine and tissues were sampled and weighed. Testes were used for histological analysis and BTB assay and to determine daily sperm production (DSP). Epididymides were used to evaluate sperm parameters.

### Histological Analysis

For histological analysis, testes were removed and fixed in Bouin solution, and the other tissue samples were fixed in 10% formalin. Dehydration was carried out at room temperature using ascending concentrations of ethanol and shifting to xylene. After clearing, tissues were embedded in paraffin wax and 3- to 5-μm-thick sections were cut using a microtome (Thermo Fisher Scientific, UK). Sections were transferred to albumenized slides that were preheated to 37°C. Tissues were rehydrated in descending concentrations of ethanol, stained with hematoxylin/eosin and covered with a coverslip. The prepared slides were observed under an Eclipse 50i microscope (Nikon Instruments Inc., Tokyo, Japan) equipped with a digital camera (Canon, Japan). For TUNEL assay, testicular sections were revealed with In Situ Cell Death Kit (Roche Applied Science, Indiana, USA) as previously described (25).

### Intratesticular and Serum Testosterone Determination

Testosterone was extracted from PND 31 testis homogenates with diethyl ether followed by evaporation of the organic phase and reconstitution of extracted testosterone in 0.1% PBS. Serum testosterone of PND 90 animals were also evaluated. Testosterone concentration was measured with an electrochemiluminescence assay (Roche Diagnostics, Mannheim, Germany) using a Cobas e 411 analyzer according to the manufacturer’s instructions. Testosterone assay sensitivity was 10 ng/dl, and intra and interassay CV were 2.4% and 2.6%, respectively.

### Thiobarbituric Acid Reactive Substances Assay

Lipid oxidation was determined by the colorimetric assay of TBARS (26). Testis homogenates were performed in PBS containing 0.4% w/v butylated hydroxytoluene on ice and then disrupted by ultrasonic irradiation. An aliquot (25 µl, corresponding to 450 µg protein) was added to 175 µl mixed reaction solution (0.15% w/v SDS, 0.5 N HCl, 0.75% w/v phosphotungstic acid, and 0.175% w/v 2-thiobarbituric acid). The mixture was heated in a boiling water bath for 45 min. TBARS were extracted with 200 µl of n-butanol. After a centrifugation at 10,000*g* for 5 min at 4°C, the absorbance at 532 nm of the butanolic phase was measured. A calibration curve was performed using malondialdehyde (MDA), generated from 1,1,3,3-tetramethoxypropane (0.4–8 µM), as standard to express the absorbance changes as nmol MDA/µg protein.

### Blood–Testis Barrier Integrity Assay

The permeability of the BTB was assessed with a biotin tracer as described previously by Perez et al (27). Immediately after testes isolation, a solution of 10 mg/ml EZ-Link Sulfo-NHS-LC-Biotin (Pierce) dissolved in PBS containing 1 mM CaCl_2_ was injected into the testis. The administered volume represented 10% of testis weight. Testes were then incubated at 34°C for 30 min, immersed in 4% paraformaldehyde and embedded in paraffin. For localization of the biotin tracer, testis sections (5 µm thick) obtained from different levels were deparaffinized and hydrated. To avoid nonspecific staining, sections were blocked with 5% nonfat dry milk in PBS containing 0.01% Triton X-100 for 15 min prior to incubation with streptavidin-rhodamine (1:300; Invitrogen, USA) for 45 min at room temperature. After nuclear staining with DAPI, sections were mounted in buffered glycerin and observed by Ahiophot fluorescent microscope with epi-illumination. At least 50 seminiferous tubules from 3 nonconsecutive testis sections from each rat were examined. Results are expressed as % of permeable tubules.

### RT-qPCR Analysis

Total RNA was isolated from testis homogenates with TRI Reagent (Sigma-Aldrich). The amount of RNA was estimated by spectrophotometry at 260 nm. RT was performed on 2 µg RNA at 42°C for 50 min with a mixture containing 200 U MMLV reverse transcriptase enzyme, 125 ng random primers and 0.5 mM dNTP Mix (Invitrogen, Argentina).

qPCR was performed by a Step One Real Time PCR System (Applied Biosystems, Warrington, UK). The specific primers for RT-qPCR are shown in **Table 1**. Amplification was carried out as recommended by the manufacturer: 25 µl reaction mixture containing 12.5 µl of SYBR Green PCR Master mix (Applied Biosystems), the appropriate primer concentration and 1 µl of cDNA. The relative cDNA concentrations were established by a standard curve using sequential dilutions of a cDNA sample. The amplification program included the initial denaturation step at 95°C for 10 min, 40 cycles of denaturation at 95°C for 15 s, and annealing and extension at 60°C for 1 min. Fluorescence was measured at the end of each extension step. After amplification, melting curves were acquired and used to determine the specificity of PCR products. The relative standard curve method was used to calculate relative gene expression. Relative mRNA levels were normalized to the reference genes HPRT1 and βactin.

**Table 1:** Rat-specific primers sets for analysis by RT-qPCR.

Gene	Primer sequence	Product size (PB)	Accession number
**AR**	FWD: 5′-GTGAAATGGGACCTTGGATG-3′	103	NM_012502.1
	REV: 5′-GGTGGGAAGTAATAGTCGATGG-3′		
**Claudin11**	FWD: 5′-TGGTCTCTACCACTGCAAGC-3′	95	NM_053457.2
	REV: 5′-CCAGAACTGAGGCAGCAATC-3′		
**Occludin**	FWD: 5′-CCACTATGAAACCGACTACACG-3′	73	NM_031329.2
	REV: 5′-ATATTCCCTGAGCCAGTCCTC-3′		
**ZO-1**	FWD: 5′-CATCTAAACCTCCAAGTGCTTC-3′	132	NM_001106266.1
	REV: 5′-CAATATCTTCAGGTGGCTTCG-3′		
**Connexin43**	FWD: 5′-ACTTCAGCCTCCAAGGAGTTC-3′	79	NM_012567.2
	REV: 5′- ATGTCTGGGCACCTCTCTTTC-3′		
**Connexin46**	FWD: 5′-GTCACTGGTGCTCAACATGC-3′	85	NM_024376.2
	REV: 5′-ATCTGGGTTGAAGTGGTTGG-3′		
**Connexin50**	FWD: 5′-CGGCAGAGTTTGGCTCAC-3′	144	NM_153465.2
	REV: 5′-GCCTCATCATAGCAGACGTTC-3′		
**HPRT1**	FWD: 5′-AGTTCTTTGCTGACCTGCTG-3′	127	NM_012583.2
	REV: 5′-TTTATGTCCCCCGTTGACTG-3′		
**β-actin**	FWD: 5′-TGGCACCACACTTTCTACAAT-3′	189	NM_031144.3
	REV: 5′-GGTACGACCAGAGGCATACA-3′		

FWD, forward; REV, reverse.

### Determination of Daily Sperm Production

At PND 90, testes were collected and processed following the experimental procedure described in Fernandes et al (28). The tunica albuginea was removed from one testis and the parenchyma was homogenized in 0.9% w/v NaCl containing 0.5% w/v Triton X100. Then, the homogenates were ultrasonic disrupted for 30 s. Samples were diluted at 1:10 and transferred to a Neubauer chamber and counted in quadruplicate. Elongated spermatid nuclei with a shape characteristic of step 19 spermatids and resistant to homogenization were counted to determine the number of elongated spermatid nuclei. To calculate the DSP, the number of spermatids was divided by 6.1, which is the number of days of the seminiferous cycle in which these spermatids are present in the seminiferous epithelium (29).

### Assessment of Sperm Parameters

At PND 90, sperm were recovered from cauda epididymis. The epididymides were placed in a conical tube, covered with 750 µl of fresh medium (30) and sperm were allowed to swim up at 37°C. After 10 min, aliquots of the upper sperm layer were recovered for motility and morphology evaluation. To evaluate total motility (progressive + nonprogressive), sperm suspensions were placed on pre-warmed slides and analyzed subjectively under a light microscope (400× magnification). To assess sperm morphology, a 10 µl aliquot of the sperm suspension was smeared over a microscope slide. After drying in air, the smear was fixed with methanol for 5 min, washed with distilled water, stained with Harris hematoxylin for 15 min, and finally washed with tap water. Sperm morphology was evaluated using a Nikon microscope (Nikon Instruments Inc., Melville, NY, USA) at 1000× magnification. In all cases, at least 200 spermatozoa from each sample were assessed.

### Statistical Analysis

At least five animals per treatment group were used, and data were presented as mean ± SD. The variables under study were first submitted to tests of normal distribution (Shapiro-Wilk’s Test) and for homogeneity of variances (Levene’s Test). Then, one-way ANOVA followed by Tukey-Kramer test for the comparison of multiple groups was performed. To assume normal distribution, percentages were expressed as ratios and subjected to the arcsine square root transformation. Results were compared using the unpaired Student t test. Probabilities <0.05 were considered statistically significant. InfoStat 2016 (Grupo InfoStat, Facultad de Ciencias Agropecuarias, Universidad Nacional de Córdoba, Argentina) was used.

## Results

### Glyphosate and Roundup Effects on Serum Biomarkers and Testis and Body Weight

Initially, rats were exposed to low doses (2 and 50 mg/kg/day) of G or R from PND 14 to 30, a period of life that is essential to complete a functional BTB. Serum biomarkers for renal (urea, creatinine) and liver (aspartate and alanine aminotransferases: AST and ALT) function were analyzed. These biomarkers did not change in treated groups compared with the control (**Table 2**). In addition, treatments did not alter liver, kidney, stomach, and intestine histology (data not shown). To explore the impact of G or R exposure on male reproductive system we assessed body and testicular weights after treatments. No differences in body and testis weight and in the ratio testis/body weight at any G or R dose tested were observed (**Table 2**).

**Table 2:** Effect of G and R treatment on serum biochemical markers and on body weight, testis weight, and relative testis weight.

	Control	G 2 mg/kg/day	G 5O mg/kg/day	R 2 mg/kg/day	R 50 mg/kg/day
**Urea (mg/dl)**	31.5 ± 3.3	34.9 ± 4.2	33.0 ± 7.1	32.0 ± 5.7	35.9 ± 6.2
**Creatinine (mg/dl)**	0.13 ± 0.09	0.12 ± 0.05	0.18 ± 0.11	0.17 ± 0.04	0.21 ± 0.03
**AST (UI/l)**	134 ± 18	128 ± 13	136 ± 15	142 ± 16	111 ± 12
**ALT (UI/l)**	37.8 ± 7.0	37,9 ± 6,4	35.0 ± 5.3	37.2 ± 7.8	33.0 ± 8.2
**Body Weight(g)**	112.4 ± 11.2	101.1 ± 12.7	111.3 ± 17.0	105.3 ± 12.5	112.8 ± 13.0
**Testis Weight (mg)**	375.7 ± 90.6	327,7 ± 53,4	366.9 ± 80.2	332.3 ± 83.8	348.9 ± 77.4
**TW/BW Ratio (%)**	0.33 ± 0.05	0.32 ± 0.03	0.33 ± 0.03	0.31 ± 0.06	0.31 ± 0.04

Animals (n = 5/group) were treated with 2 and 50 mg/kg/day of G or R from PND 14 to 30. At PND 31, body and testicular weights were assessed, and blood was sampled by intracardiac puncture to determine serum parameters. Results are presented as mean ± SD.

### Glyphosate and Roundup Effects on Testicular Histology and Blood–Testis Barrier Function

**Figure 2:**
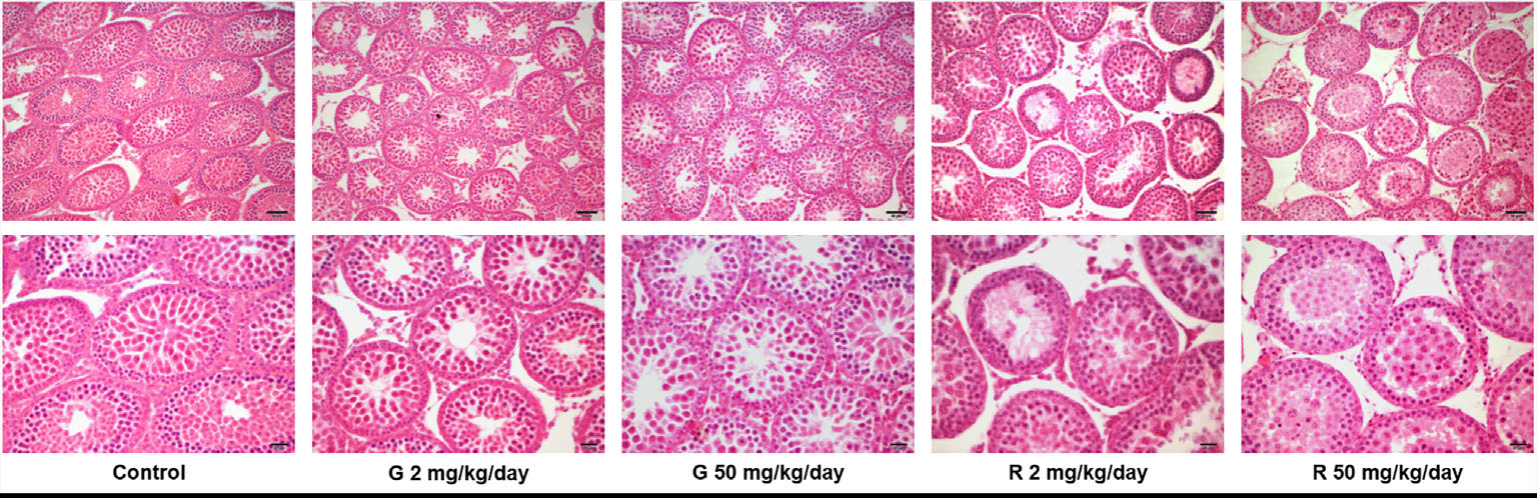
Effect of G and R treatment on testicular histology. Animals (n = 5/group) were treated with 2 and 50 mg/kg/day of G or R from PND 14 to 30. At PND 31, animals were euthanized, and testes were removed and fixed in Bouin solution. Sections (3–5 μm) obtained from the poles and equatorial areas were stained with hematoxylin/eosin and examined by light microscopy. Representative pictures are shown. Upper panels: scale bar, 50 µm. Higher magnifications images are shown in lower panels: scale bar, 10 µm.

Histological examination of the testes revealed differences in the seminiferous tubules among control and treated groups (**Figure 2**). Some seminiferous tubules of G2, G50, and R2 groups showed a disorganized epithelium, with an apparent low cellular adhesion. In R50 treated males, tubules presented severe disorganization and epithelial desquamation of the most differentiated cells (spermatocytes and round spermatids). In this group, the percentage of affected tubules is higher than in the other groups tested. Although epithelium disorganization was observed in treated groups, the histological characteristics of Sertoli cells -nucleus located parallel to basement membrane next to spermatogonia and premeiotic spermatocytes and triangular in shape- remained unaltered. Additionally, TUNEL analysis of rat testis section from control and R50 treated animals were performed. Only a few seminiferous tubules with some TUNEL positive cells located in areas not corresponding to positions occupied by Sertoli cells were observed in both groups (**Supplementary Figure 1**).

**Figure 3:**
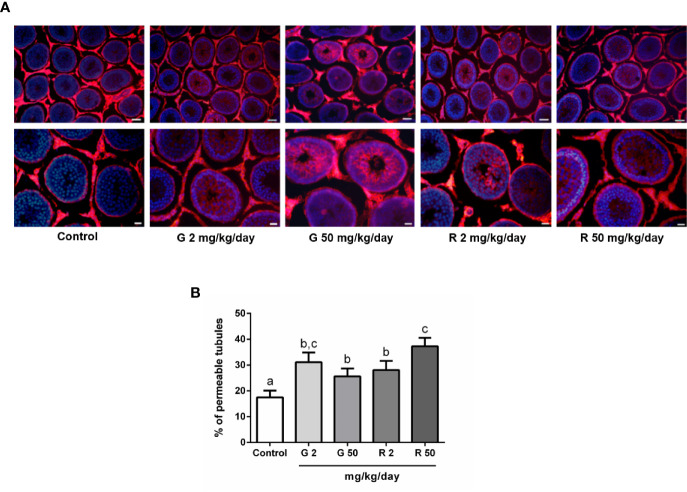
Effect of G and R treatment on BTB permeability. Animals (n=7/group) were treated with 2 and 50 mg/kg/day of G or R from PND 14 to 30. At PND 31, animals were euthanized, and testis were dissected and used for BTB integrity assay. Testes were injected with a biotin tracer (red) and cell nuclei were dyed with 4′-6-diamidino-2-phenylindole (DAPI, blue). **(A)** Representative pictures are shown in upper panels. Scale bar, 50µm. Higher magnifications images are shown in lower panels. Scale bar, 10 µm. **(B)** Quantification of permeable tubules in each experimental group. At least 50 seminiferous tubules from three nonconsecutive testis sections from each rat were examined. Results are presented as mean ± SD, different letters indicate statistically significant differences P<0.05.

To analyze the effects of G or R treatment on BTB integrity, the permeability of the BTB was evaluated using a biotin tracer which is excluded from the adluminal compartment of intact seminiferous tubules. **Figure 3A** shows that the tracer entered the adluminal compartment in animals treated with G or R at both doses tested. **Figure 3B** shows the data obtained after determining the percentage of permeable tubules in the different experimental groups. A significant increase in the percentage of seminiferous tubules with a permeable barrier in G and R groups was observed.

**Figure 4:**
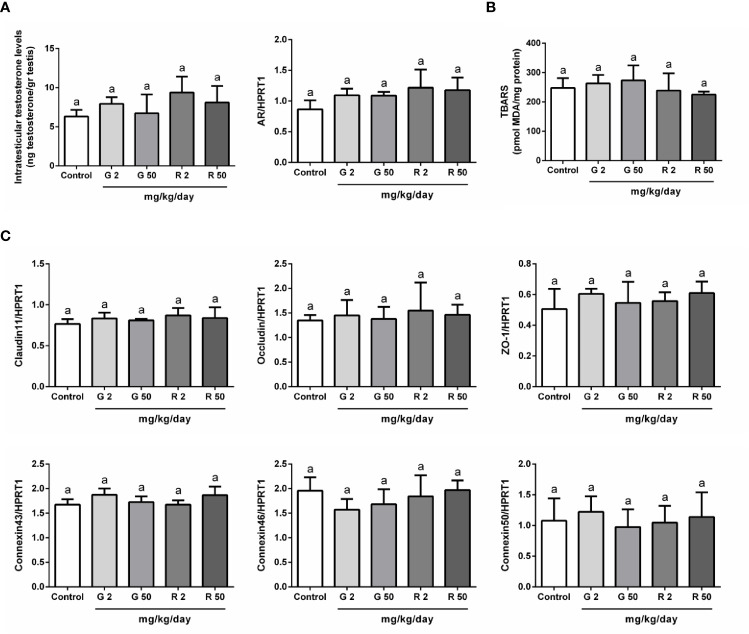
Analysis of possible mechanisms involved in G and R effects on BTB integrity. Animals (n=5/group) were treated with 2 or 50 mg/kg/day of G or R from PND 14 to 30. At PND 31, animals were euthanized, and testes were homogenized to evaluate **(A)** intratesticular testosterone and **(B)** Thiobarbituric Acid Reactive Substances (TBARS). Additionally, testes were used to evaluate mRNA levels of genes encoding for **(A)** Androgen Receptor (AR) and **(C)** BTB proteins (claudin11, occludin, ZO-1, connexin43, 46, and 50). HPRT1 was used as internal control. Results are presented as mean ± SD, different letters indicate statistically significant differences P<0.05.

The next set of experiments was performed to evaluate possible mechanisms that would explain the deleterious effects of G or R treatments on BTB permeability. First, as testosterone is the main regulator of BTB formation and integrity, intratesticular testosterone (ITT) levels and androgen receptor (AR) expression were evaluated. **Figure 4A** shows that G and R treatments did not modify either ITT levels or AR expression. Secondly, as it has been demonstrated that reactive oxygen species (ROS) mediate some of the harmful effects in BTB integrity, we decided to evaluate TBARS levels after G or R treatments. **Figure 4B** shows that TBARS levels were not modified by G or R exposure. Thirdly, the expression of intercellular junction proteins such as claudin11, occludin, ZO-1, connexin43, 46, and 50 which are components of the BTB, was analyzed. **Figure 4C** shows that G or R treatments did not modify claudin11, occludin, ZO-1, connexin43, 46, and 50 mRNA levels.

### Juvenile Roundup Treatment Effects on Adult Animals

In order to evaluate possible consequences of juvenile herbicide treatment in adulthood, a set of animals was treated with 50 mg/kg/day of R from PND 14 to 30 and then allowed to grow until PND 90. Several parameters were analyzed at this age. As it was observed in PND 31, **Table 3** shows that no differences in urea, creatinine, AST, and ALT levels between groups were observed.

**Table 3:** Effect of juvenile R treatment on adult serum biochemical markers and on body, testis, and epididymal weight and relative testis and epididymal weight.

	Control	R 50 mg/kg/day
**Urea (mg/dl)**	46.4 ± 3.3	49.2 ± 8
**Creatinine (mg/dl)**	0.30 ± 0.09	0.29 ± 0.05
**AST (UI/l)**	99.4 ± 17.5	87.9 ± 28.1
**ALT (UI/l)**	33.8 ± 3.4	29.9 ± 2.2
**Body Weight**	577.3 ± 54.6	596.7 ± 18.0
**Testis Weight**	1.9 ± 0.2	1.8 ± 0.3
**TW/BW Ratio (%)**	0.36 ± 0.03	0.34 ± 0.02
**Epididymal Weight (g)**	0.94 ± 0.30	0.85 ± 0.30
**EW/BW Ratio (%)**	0.16 ± 0.05	0.14 ± 0.05
**Testosterone levels (ng/dl)**	195 ± 17	189 ± 22

Animals (n = 5/group) were treated with 50 mg/kg/day of R from PND 14 to 30. At PND 90, body, testicular, and epididymal weights were assessed and blood was sampled by intracardiac puncture to determine serum parameters. Results are presented as mean ± SD.

**Figure 5:**
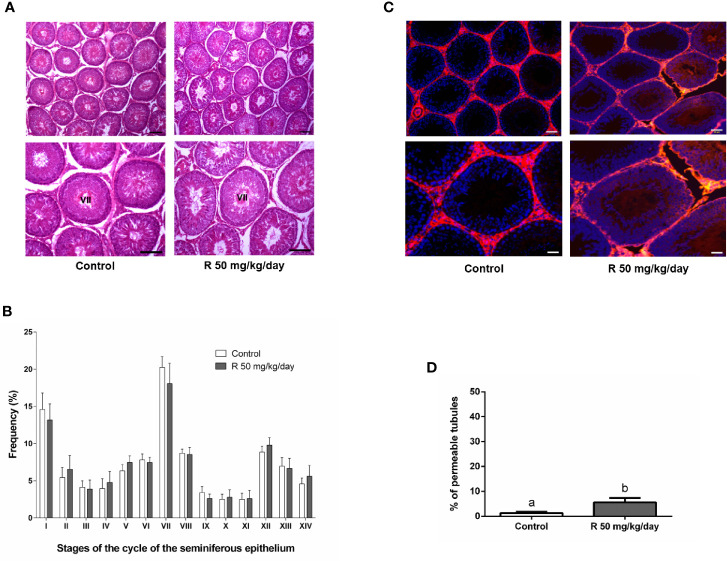
Effect of juvenile R treatment on adult testicular histology and BTB integrity. Animals (n = 7/group) were treated with 50 mg/kg/day of R from PND 14 to 30. At PND 90, animals were euthanized. **(A)** Testes were removed and fixed in Bouin solution. Sections (3–5 μm) obtained from the poles and equatorial areas were stained with hematoxylin/eosin and examined by light microscope. Representative pictures are shown. Higher magnification images are shown in lower panels. Scale bar, 50 µm. **(B)** Quantitative analysis of the frequency of the stages of the cycle of the seminiferous tubule epithelium in each experimental group. The stages are indicated by roman numerals I–XIV and expressed as percentage. Results are presented as mean ± SD. No statistically significant differences were found between Control and R 50 mg/kg/day group. **(C)** Testes were injected with a biotin tracer (red) and cell nuclei were dyed with 4′-6-diamidino-2-phenylindole (DAPI, blue). Representative pictures are shown in upper panels. Scale bar, 50 µm. Higher magnification images are shown in lower panels. Scale bar, 10 µm.**(D)** Quantification of permeable tubules in each experimental group. At least 50 seminiferous tubules from three nonconsecutive testis sections from each rat were examined. Results are presented as mean ± SD, different letters indicate statistically significant differences P < 0.05.

**Figure 6:**
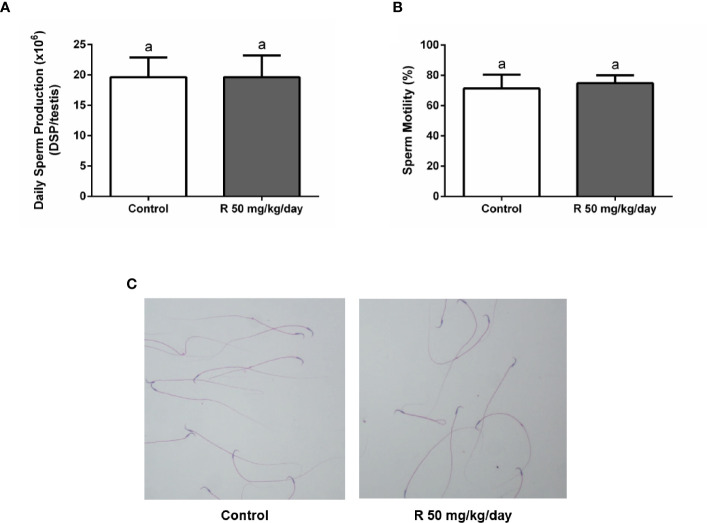
Effect of juvenile R treatment on daily sperm production (DSP) and sperm parameters. Animals (n = 7/group) were treated with 50 mg/kg/day of R from PND 14 to 30. At PND 90, **(A)** testes were collected to determine DSP; **(B, C)** epididymal sperm were used to analyze sperm motility and morphology. **(A, B)** Results are presented as mean ± SD, different letters indicate statistically significant differences P < 0.05. **(C)** Representative microphotographs are shown.

**Figure 5A** shows the histological examination of testis at PND 90 in control and R50 groups. Tubules had normal cellular associations and complete spermatogenesis. **Figure 5B** shows the analysis of the frequency of the stages of the seminiferous epithelium. No alteration of the presence of the different stages of the cycle of the seminiferous epithelium was found between groups. **Figure 5C** shows the study with the biotin tracer and **Figure 5D** shows the data obtained after determining the percentage of permeable tubules. A small increase in the percentage of permeable tubule in R50 group was observed. Despite this small increase in BTB permeability, testicular weight, and DSP were not modified by juvenile treatment with R (**Table 3** and **Figure 6A**).

Cauda epididymal sperm were used to analyze sperm motility and morphology. No differences in motility and morphology were observed in sperm of both groups (**Figures 6B, C**). In addition, no changes were observed in epididymal weight and in epididymal/body weight ratio between groups (**Table 3**).

## Discussion

Mounting evidence indicates a declining trend in the male reproductive health of both wildlife and humans. A meta-analysis reporting a significant decline in sperm counts between 1973 and 2011 among men unselected by fertility from North America, Europe, Australia, and New Zealand (31) arose considerable scientific and public concern regarding the adverse effects of various environmental contaminants on male reproduction. Thus, several studies were conducted to assess the impact of xenobiotics on male reproductive health. In this context, the juvenile population requires special attention due to a higher sensitivity to xenobiotic exposure (32). Furthermore, evidence shows the impact of pollutants on development and reproductive functions (33–35). Notwithstanding the foregoing, no studies are available regarding the consequence of G or R exposure during critical periods of the male reproductive development, such as when a functional BTB is established. Therefore, the objective of our work was to evaluate the impact of low doses of G and R exposure of juvenile rats on the assembly of BTB and its possible influence on adult life.

The BTB provides a unique microenvironment in the adluminal compartment of the seminiferous epithelium throughout the isolation of postmeiotic germ cells from blood stream and its capacity to regulate the entry of substances (36). Any imbalance in the composition of the adluminal compartment fluid, as a consequence of BTB damage, leads to impaired spermatogenesis. The relationship between BTB disfunction and impediment of meiosis and spermatogenesis was widely documented along the years. In this context, several experimental approaches which caused BTB disruption result in disturbance of seminiferous epithelium homeostasis and ultimately loss of spermatogenesis. Among them, it is relevant to highlight the analysis of knockout mice for genes encoding claudin11 (37) and occludin (38), which despite displaying variable phenotype, in these models it is observable that spermatogenesis does not proceed. In addition, rats with autoimmune orchitis display loss of occludin expression and consequently an increment in BTB permeability associated with damage of seminiferous epithelium and disruption of spermatogenesis (27). Although the reason for declining spermatogenesis during aging remains largely unknown, it is suspected that impaired function of the BTB might account for it (39). It is also worth mentioning that environmental toxicants, such as cadmium and bisphenol A, induce BTB disruption eliciting subsequent damage to germ-cell adhesion, thereby leading to germ-cell loss, reduced sperm count, and male infertility or subfertility (40–42). Overall, there is a consensus that BTB impairment leads to spermatogenesis arrest.

As for the evaluation performed on PND 31 rats, biochemical markers of hepatic and renal function were determined as indicators of toxicity. Several studies have shown altered hepatic biochemical parameters using different models of doses and exposition however, the majority of them performed the analysis administrating high doses which lack of toxicological relevance (43, 44). However, under our experimental conditions G or R treatments did not affect ALT and AST levels. In addition, body, testis, and relative testis weight were not affected by the treatments; nevertheless, the lack of effect on organ weight should not be used to neglect other biological effects on testis that may be more sensitive to the toxicants. To this respect, it is worth mentioning that several changes in histological structure of rat testis after G or R treatments from PND 23 for 35 days have been previously observed. Nardi et al (45) showed a decrease in Sertoli and Leydig cell number and an increase in the percentage of degenerated Sertoli and Leydig cells after daily exposure of juvenile rats to 50 and 100 mg/kg of G. Moreover, Romano et al (10) showed an increase in luminal diameter of seminiferous tubules and a reduction in seminiferous epithelium after 5, 50, or 250 mg/kg/day of R treatment. In the present study, testicular histological analysis of juvenile animals exposed to doses of 2 and 50 mg/kg/day of herbicides from PND 14 to PND 30 showed a disorganized epithelium with an apparent low cellular adhesion, effects that are more pronounced when animals were exposed to 50 mg/kg/day of R. Although epithelium disorganization was observed in treated groups, the histological characteristics of Sertoli cells remained unaltered. In addition, Sertoli cell apoptosis was not observed.

As mentioned above, it is well known that the BTB of adult and juvenile individuals is a target of multiple xenobiotics (19, 46–50). In our study BTB impairment, demonstrated by the significant increase in BTB permeability in both G- and R-treated groups, has been observed. This alteration occurred in both G- and R-treated groups at both doses tested. These results suggest that BTB function of juvenile rats may result injured after exposure to low doses of the herbicide.

Androgens are crucial to establish and maintain BTB integrity (51–53). In this context, it is tempting to speculate that G or R effects on BTB integrity can be partially attributed to deleterious effects on androgen action―testosterone production and/or androgen receptor expression. In the present study, no significant differences among groups in intratesticular testosterone levels were observed. Androgen receptor has also been postulated as a target for the deleterious effects of G or R, however, results are somehow controversial. On the one hand, it has been shown that treatment of drakes with R decreases androgen receptor expression in Sertoli cells (54). On the other hand in rats, it has been observed that G treatment does not modify Sertoli cell androgen receptor expression (55). The results presented herein show that G or R treatments did not modify testicular androgen receptor mRNA levels under our experimental conditions. Altogether, these results suggest that testosterone levels or androgen receptor expression are not possibly responsible for the observed effects of G or R on BTB integrity in our experimental model.

An additional aspect to be considered is related to the growing evidence indicating that exposure to different classes of environmental toxicants commonly increases oxidative stress in the testis (56–60). As it has been observed that G and R increase oxidative stress in rat liver (61, 62), we then hypothesized that herbicides might cause a disturbance in redox balance that could be responsible for the alteration in BTB permeability. However, the results presented herein suggest that it might not be the case as G and R treatment did not modify TBARS levels.

Cell junction proteins have been regarded as early targets for different classes of reproductive toxicants (48, 63). To this respect, a decrease in the expression of junction proteins that participate in BTB formation after exposure to several toxicants has been demonstrated (47, 59, 64). Therefore, we decided to analyze occludin, claudin11, and ZO-1 as representative tight junction proteins and connexin43, 46, and 50 as representative gap junction proteins of BTB. In this regard, we observed that G or R treatments did not modify the expression of these junction proteins suggesting that the increase of BTB permeability may not be attributed to a reduction in their expression. Delocalization of claudin11 after herbicide treatment *in vitro* has been previously observed and would explain, at least in part, the effects of G and R in the integrity of BTB (20).

Bearing in mind that Sertoli cell apoptosis, TBARS and intratesticular testosterone levels, and androgen receptor and junction protein expression remained unchanged among experimental groups, additional experiments will be necessary to determine how G and R alter BTB permeability.

In order to determine whether herbicide exposure in juvenile animals has any effect on adult reproductive function, a group of animals treated with 50 mg/kg/day of R from PND 14 to PND 30 was evaluated on PND 90. Testicular architecture was apparently recovered at PND 90 and no alteration of the frequency of the stages of the cycle of the seminiferous tubule epithelium was observed between groups. When evaluating BTB function, an increase in BTB permeability was observed in treated animals, however, the DSP and testicular weight were not modified. Finally, as reduction in sperm motility after direct G or R treatment had been demonstrated (65–68), we decided to analyze epididymal sperm parameters. In the study presented herein R did not change epididymal sperm morphology and motility. Altogether, these results suggest that the alterations observed after treatment during the juvenile period turn out to be reversible and do not modify adult sperm production, morphology, and motility.

The use of glyphosate as an herbicide continues to expand and the doses declared as safe must be continually re-evaluated. Although the EPA establishes that a dose of 1 mg/kg/day is safe and harmless, our results call to be cautious about this statement. The results presented herein suggest that toxic effects may appear even with doses declared safe by EPA. In addition, even though the effects of exposure in juvenile stages seem to be reversible, more studies will be necessary to determine what happens to the permeability of BTB if exposure continues into adulthood. On the other hand, the reversible nature of the effects may enable, if these effects take place in humans, to apply strategies for the exposed populations: early detection and removal of exposure sources and/or migration of exposed inhabitants from contaminated areas.

In conclusion, the results presented herein show that continuous exposure to low doses of G or R alters BTB permeability in juvenile rats. Considering that DSP in adult animals, which have been unexposed to the herbicide for a prolonged period, is indistinguishable from that in control animals, it is feasible to think that BTB impairment is a reversible phenomenon. These results warrant further investigation of glyphosate-mediated reproductive damage of human juvenile populations exposed to low doses of G or R. Such analysis may include the determination of follicle stimulating hormone (FSH), luteinizing hormone (LH), anti-Müllerian hormone (AMH), inhibin B, and testosterone serum levels to get insight into Sertoli cell maturation in juvenile people as well as semen analysis in adult population.

## Data Availability Statement

The raw data supporting the conclusions of this article will be made available by the authors, without undue reservation.

## Ethics Statement

The animal study was reviewed and approved by Comité Institucional de Cuidado y Uso de Animales de Laboratorio (CICUAL) from the Hospital de Niños Ricardo Gutiérrez (Res #2018-002).

## Author Contributions

SM, MG, and MR conceived and designed the experiments. AG, GR, CC, DC, CM-B, and CS performed the experiments. SM, MG, CM-B, and DC analyzed the data. EP, MC, and CS contributed reagents/materials/analysis tools. SM, MG, MR, and AG wrote the paper. All authors contributed to the article and approved the submitted version.

## Funding

This work was supported by the Agencia Nacional de Promoción Científica y Tecnológica (PICT 2018/1291; PICT2015/228) and the Consejo Nacional de Investigaciones Científicas y Técnicas (CONICET) (PIP 2015/127).

## Conflict of Interest

The authors declare that the research was conducted in the absence of any commercial or financial relationships that could be construed as a potential conflict of interest.

## Supplementary Material

Supplementary Figure 1 Effect of R treatment on testicular apoptosis. Animals (n=3/group) were treated with 50 mg/kg/day of R from PND 14 to 30. At PND 31, animals were euthanized, and testes were removed. Testis sections (3-5 mm) were used for TUNEL analysis. Representative photomicrographs of TUNEL assay are shown. Arrowheads indicate TUNEL-positive cells (green). Cell nuclei were dyed with Hoechst (blue). Scale bar, 10 μm.
