# Supplementary figures and images for: Low Doses of Glyphosate/Roundup Alter Blood–Testis Barrier Integrity in Juvenile Rats
Source: Front Endocrinol (Lausanne). 2021 Mar 11;12:615678. doi: 10.3389/fendo.2021.615678 (PMC7992013; doi:10.3389/fendo.2021.615678)

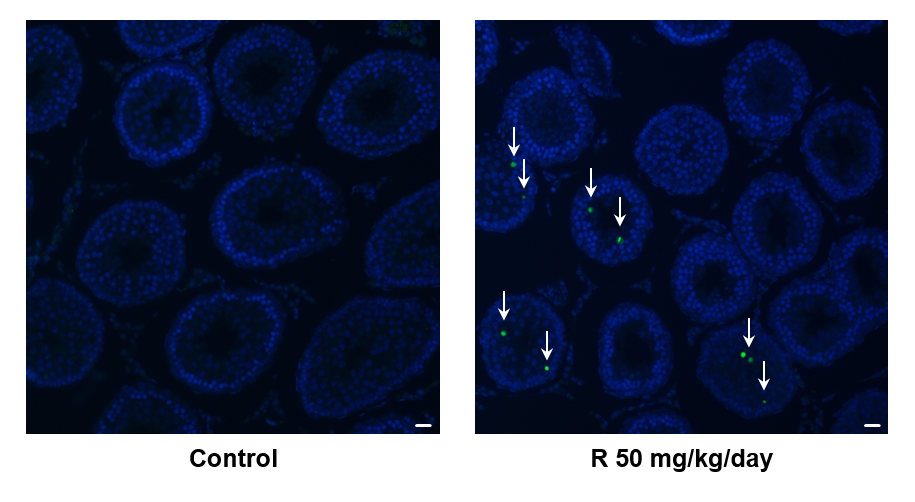

Supplement: Supplementary Figure 1 — Effect of R treatment on testicular apoptosis. Animals (n=3/group) were treated with 50 mg/kg/day of R from PND 14 to 30. At PND 31, animals were euthanized, and testes were removed. Testis sections (3-5 mm) were used for TUNEL analysis. Representative photomicrographs of TUNEL assay are shown. Arrowheads indicate TUNEL-positive cells (green). Cell nuclei were dyed with Hoechst (blue). Scale bar, 10 μm. [file Image_1.tif]
